# Using vascular biomarkers to assess heart failure event risk in hospitalized patients with and without AKI

**DOI:** 10.1186/s12882-025-04169-1

**Published:** 2025-06-02

**Authors:** Audrey A. Shi, Anna Simone Andrawis, Aditya Biswas, Francis P. Wilson, Wassim Obeid, Heather Thiessen Philbrook, Alan S. Go, T. Alp Ikizler, Edward D. Siew, Vernon M. Chinchilli, Chi-Yuan Hsu, Amit X. Garg, W. Brian Reeves, David K. Prince, Pavan Bhatraju, Steve G. Coca, Kathleen D. Liu, Paul L. Kimmel, James S. Kaufman, Mark W. Wurfel, Jonathan Himmelfarb, Chirag R. Parikh, Sherry G. Mansour, for the ASSESS-AKI Consortium*

**Affiliations:** 1 Department of Internal Medicine, Section of Nephrology, Yale School of Medicine, New Haven, CT USA; 2 Clinical Translational Research Accelerator, Yale University School of Medicine, New Haven, CT USA; 3 Johns Hopkins University, Baltimore, MD USA; 4 Kaiser Permanente Northern California, Pleasanton, CA USA; 5 University of California, San Francisco, San Francisco, CA USA; 6 Vanderbilt University, Nashville, TN USA; 7 The Pennsylvania State University, University Park, PA USA; 8 Western University, London, ON USA; 9 The University of Texas Health Science Center at San Antonio, San Antonio, TX USA; 10 University of Washington, Seattle, WA USA; 11 Icahn School of Medicine at Mount Sinai, New York, NY USA; 12 National Institute of Diabetes and Digestive and Kidney Diseases, Bethesda, MD USA; 13 VA New York Harbor Healthcare System, New York, NY USA

**Keywords:** Biomarkers, Vascular, Acute kidney injury, Heart failure, Hospitalized patients, Outcomes

## Abstract

**Background:**

Patients with AKI experience higher rates of heart failure (HF). This study seeks to identify criteria to assess the risk of heart failure post-hospitalization, with a special focus on AKI patients. We hypothesized that the combined use of 9 vascular biomarkers would predict future heart failure events after AKI. Using a study of 1497 hospitalized patients with and without AKI, we found that these 9 vascular biomarkers successfully stratified patients into different risk groups for HF, and were able to improve prediction of HF when added to routine clinical variables.

**Methods:**

Using the ASSESS-AKI cohort, we performed an unsupervised spectral cluster analysis with 9 plasma biomarkers measured at 3 months post-hospitalization *[Angiopoietin (angpt)-1*,* angpt-2*,* vascular endothelial growth factor (VEGF)-A*,* VEGF-C*,* VEGF-d*,* VEGF receptor 1 (R1)*,* solubleTie-2 (sTie-2)*,* placental growth factor (PlGF)*,* and basic fibroblast growth factor (bFGF)]* in 1,497 patients, half of whom had AKI. We used a Cox regression analysis to evaluate the associations between the clusters and HF. Models were adjusted for demographics, cardiovascular disease risk factors, medications, ICU status, lung disease, sepsis, clinical center, and 3-month post-discharge serum creatinine and proteinuria. We calculated change in the area under the curve (AUC) for the prediction of HF or death at 3 years by adding the biomarkers to a clinical model selected by a penalized regression with LASSO. We also calculated a net reclassification index for the addition of the biomarkers to the clinical model.

**Results:**

Three biomarker-derived clusters were identified: Cluster 1 [*n* = 302, Vascular Injury (Injury) Phenotype] had higher levels of injury markers, whereas Cluster 2 [*n* = 728, Vascular Repair (Repair) Phenotype] had higher levels of repair markers. Cluster 3 (*n* = 467) had lower levels of all markers (Dormant Phenotype). Across the entire cohort, those with the Injury Phenotype had twofold higher risk of a HF event compared to the Repair Phenotype [aHR 2.24 (95% CI: 1.57–3.19)] and noted in both participants with AKI [aHR 2.12 (95% CI: 1.35–3.34)] and without AKI [aHR 2.94 (95%CI: 1.57–5.50)]. The Dormant Phenotype was associated with higher risk of HF events only in participants without AKI. The AUC for the prediction of HF event or death at 3 years by the biomarkers was 0.76 (95% CI: 0.73–0.80), 0.77 (95% CI: 0.73–0.80) for the clinical model, and 0.80 (95% CI: 0.77–0.83) for the combined model. The addition of the biomarkers significantly improved reclassification of HF event or death.

**Conclusions:**

Vascular biomarkers can be used to derive phenotypes capable of stratifying future risk of HF events in recently hospitalized patients with or without AKI.

**Supplementary Information:**

The online version contains supplementary material available at 10.1186/s12882-025-04169-1.

## Introduction

Heart failure (HF) is the leading cause of hospitalization for patients over 65 years of age in America. It is also a major cause of death among kidney patients [1, 2]. It is well-established that combined AKI-HF patients experience higher rates of rehospitalization and early mortality. What remains unclear is the biological connections between AKI and HF—and whether patients with this interlocking condition can be identified for preventative treatment [3, 4].

Our group recently identified that blood vessel health is integral to whether AKI patients will go on to develop CKD and HF. AKI often presents with endothelial cell damage, secondary to ischemic injury [5, 6]. Such damage induces fibrosis; with vascular instability further impairing kidney function via microvascular albumin leakage [7, 14]. Vascular dysregulation of the heart induces an inflammatory cascade leading to a pro-atherogenic state, impairing vasodilation [15]. Indeed, these stated pathways of endothelial injury have been associated with later development of diastolic dysfunction characteristic of HF [16, 17].

Thus, in order to elucidate the associations between AKI and subsequent HF, we investigated the combined use of angiopoietins (Angpt-1, Angpt-2) and their soluble Tie-2 receptor (sTie2), the vascular endothelial growth factor family (VEGF A, C, D) and their main receptor (VEGFR1), placental growth factor (PlGF), and basic fibroblast growth factor (bFGF) to subgroup individuals into phenotypes, and to determine the association of these phenotypes with patient outcomes. Given the multiple known mechanisms linking endothelial dysfunction to AKI and HF, we hypothesized that hospitalized patients with elevated vascular biomarkers indicative of severe endothelial dysfunction will have a higher risk of future HF events; and that this risk would persist among AKI patients [18].

## Methods

### Study population

This is an ancillary study from the Assessment, Serial Evaluation, and Subsequent Sequelae in Acute Kidney Injury (ASSESS-AKI) cohort, which is a multicenter, matched, prospective study of hospitalized patients with and without AKI [19, 20]. Patients were matched by center; pre-admission chronic kidney disease (CKD) status; an integrated priority score based on age, prior cardiovascular disease (CVD) or diabetes, and pre-admission estimated glomerular filtration rate (eGFR); and treatment in the intensive care unit (ICU) during index hospitalization. The median follow-up after hospitalization was 4.9 years (interquartile range [IQR]: 3.6-6.0). The parent study collected blood samples at different timepoints. Our ancillary study requested blood samples from the 3-month time point after index hospitalization. We measured a panel of 9 vascular biomarkers in the blood: angpt-1, angpt-2, VEGF-A, VEGF-C, VEGF-D, VEGFR1, sTie-2, PlGF, and bFGF.

AKI at index hospitalization was defined as “≥50% relative increase and/or an absolute increase ≥ 0.3 mg/dL in the peak inpatient serum creatinine concentration compared with outpatient, non–emergency department baseline value, within 7 to 365 days before the index admission.”

No AKI was defined as < 20% relative increase or an absolute increase ≤ 0.2 mg/dL in the peak inpatient serum creatinine concentration compared with pre-admission value [20].

### Outcomes

We assessed potential associations between vascular biomarker panels (measured 3 months post-hospitalization) and the primary outcome of heart failure (HF) admissions, as defined by discharge codes for clinical HF, and confirmed via Framingham Heart Study clinical criteria ascertained from medical records [21]. Two physicians independently adjudicated all hospitalization records for HF. However, our adjudication process did not explicitly differentiate between new onset heart failure and chronic heart failure exacerbations.

We also assessed secondary outcomes of kidney disease progression (defined as “incident CKD [without pre-existing CKD at the index hospitalization, with ≥ 25% reduction in the eGFR from pre-admission eGFR, and reaching CKD stage III or worse during follow-up], or progression of preexisting CKD [eGFR < 60 ml/min/1.73 m ^2^ at the index hospitalization with ≥ 50% reduction in eGFR from pre-admission or progression to CKD Stage V or development of ESKD, or receipt of a kidney transplant”]); and all-cause mortality, defined through surveys of participants or their proxies, review of medical records, and death certificates.

### Plasma biomarkers

Samples were centrifuged at 2000 × g for 10 min at 4 °C, separated into 1-mL aliquots, and immediately stored at -80 °C. Biomarkers were measured using the Meso Scale Discovery platform (Meso Scale Diagnostics, Gaithersburg, MD), which uses electrochemiluminescence detection combined with patterned arrays for multiplex measurement. VEGF-A, VEGF-C, VEGF-D, VEGFR1, sTie-2, PlGF, and bFGF were measured using the Angiogenesis V-plex panel. Angpt-1, angpt-2 were measured using a custom created panel. All panels passed dilution-linearity and spike-recovery validation. The mean interassay coefficient of variation (CV) and the reportable range of detection for each biomarker are shown in Supplementary Tables 1 and 2. All laboratory personnel were blinded to the participants’ disease status and outcomes.

### Construction of clusters

We partitioned our patient population into subgroups based on biomarker data. The nine biomarkers of interest were log-transformed, then robust standardized. We define ‘robust standardization’ as subtraction of the median, followed by division by the median absolute deviation. These transformed data were then passed on to the Spectral Clustering algorithm without incorporation of clinical characteristics or subsequent outcomes.

The Spectral Clustering algorithm was set to use the 5 nearest-neighbors of each data point to construct its affinity matrix, and assigned cluster labels using the k-Means algorithm in the embedding space [22]. To facilitate interpretation, the number of desired clusters was set to 4. Spectral clustering is a flat clustering algorithm; and we note that similar results were obtained using the hierarchical clustering algorithm HDBSCAN. Thus, we restricted our presentation to the former.

One of our resulting 4 clusters contained only 6 patients. Due to concerns about reliability with respect to sample size, we interpreted these data points as outliers, and omitted this cluster from our analysis. The analysis was performed on Python 3.10.8 with the package scikit-learn 1.2.0.

### Statistical analysis

We stratified the biomarker concentrations by algorithm-driven clusters to identify emerging patterns. We identified three unique phenotypes, distinguished by biomarker concentrations: Vascular Injury (higher concentrations of vessel injury biomarkers: PlGF, sTie2, VEGFD, VEGFR1, and angpt-2), [23, 27] Vascular Repair (higher concentrations of vessel repair biomarkers: VEGFA, VEGFC, bFGF, and angpt-1), [28, 31] and Dormant (low levels of injury and repair biomarkers). Having defined the three clusters described above (henceforth “Injury”, “Repair”, and “Dormant”, respectively), we stratified patient clinical and demographic characteristics across clusters. We compared the variables in each stratum by using χ ^2^ and Kruskal–Wallis tests for categorical and continuous variables, respectively.

Using a Spearman’s Rank-Order correlation coefficient, we tested the correlations between the 9 biomarkers measured 3-months post-hospitalization, and displayed the results using a heat map.

Multinomial logistic regression was used to identify factors associated with cluster membership, using Cluster 2 (Repair Phenotype) as the reference group. We used a Cox proportional hazards regression model to examine associations between clusters and the primary outcome of HF, as well as secondary outcomes of kidney disease progression and all-cause mortality. We calculated hazard ratios for each outcome using Cluster 2 (Repair) as the reference group. We subsequently evaluated two multivariable models. The first model included all variables of clinical interest. The second was a Parsimonious Model, including only statistically significant variables. We assessed the proportional hazards assumption for outcomes by testing weighted Schoenfeld residuals.

Variables in the full model included: demographics (age, sex, and race/ethnicity); CVD risk factors (smoking, hypertension, diabetes mellitus, body mass index, history of CVD, HF, CKD); medications (vasopressors, renin angiotensin aldosterone system inhibitors, diuretics, and non-steroidal anti-inflammatory drugs); hospitalization in the ICU; chronic lung disease; chronic obstructive pulmonary disease; sepsis; serum creatinine and urine protein at time of biomarker measurement (3 months post-hospitalization); and admission center. The Parsimonious Model included: demographics (age, sex, and race/ethnicity); CVD risk factors (hypertension, diabetes mellitus, history of CVD, HF, CKD); hospitalization in the ICU; chronic obstructive pulmonary disease; sepsis; serum creatinine and urine protein at time of biomarker measurement (3 months after hospitalization); and admission center. No imputations were done for missing covariates, because rates of missingness were minimal.

For primary outcome of HF, we also performed an analysis adjusting for N terminal-pro hormone b-type natriuretic peptide (NT-proBNP) and troponin measurements at 3 months, in addition to the fully-adjusted model variables. We accounted for the competing risk of death with outcome of HF by creating a composite outcome of HF or death.

To assess the predictive power of the biomarkers, we calculated the area under receiver operating characteristic (ROC) curve for the clinical model (which was selected by performing a penalized regression with LASSO and included the following variables: demographics [age, gender, race, ethnicity]; CKD and CHF at baseline; baseline eGFR; BMI, serum creatinine, and urine protein at 3 months; smoker status; COPD; CVD; sepsis; diabetes; ICU status; and admission center); the vascular biomarkers model; and a combined biomarker-clinical model, with a combined outcome of HF or death at 3 years. Significance in AUC change was calculated using the DeLong test. Analyses for ROC curves were performed across the entire cohort, as well as stratified by AKI status. Net reclassification index (NRI) was calculated for the additional benefit of the biomarkers to the clinical model. NRI was calculated using continuous reclassification, defined as any upward or downward movement in predicted risk between models, regardless of category boundaries. The equation used to calculate reclassification of events sand non-events is as follows: NRI_event_= (Up_event_ − Down_event_) / Total events and NRI_non_-_event_= (Up_non−event_ – Down_non−event_) / Total non-events.

Statistical analyses were conducted using SAS version 9.4 (SAS Institute, Inc, Cary, NC).

The Institutional Review Boards of the Data Coordinating Center and participating Clinical Research Centers’ institutions approved the study.

## Results

### Biomarker-derived clusters

**Fig. 1:**
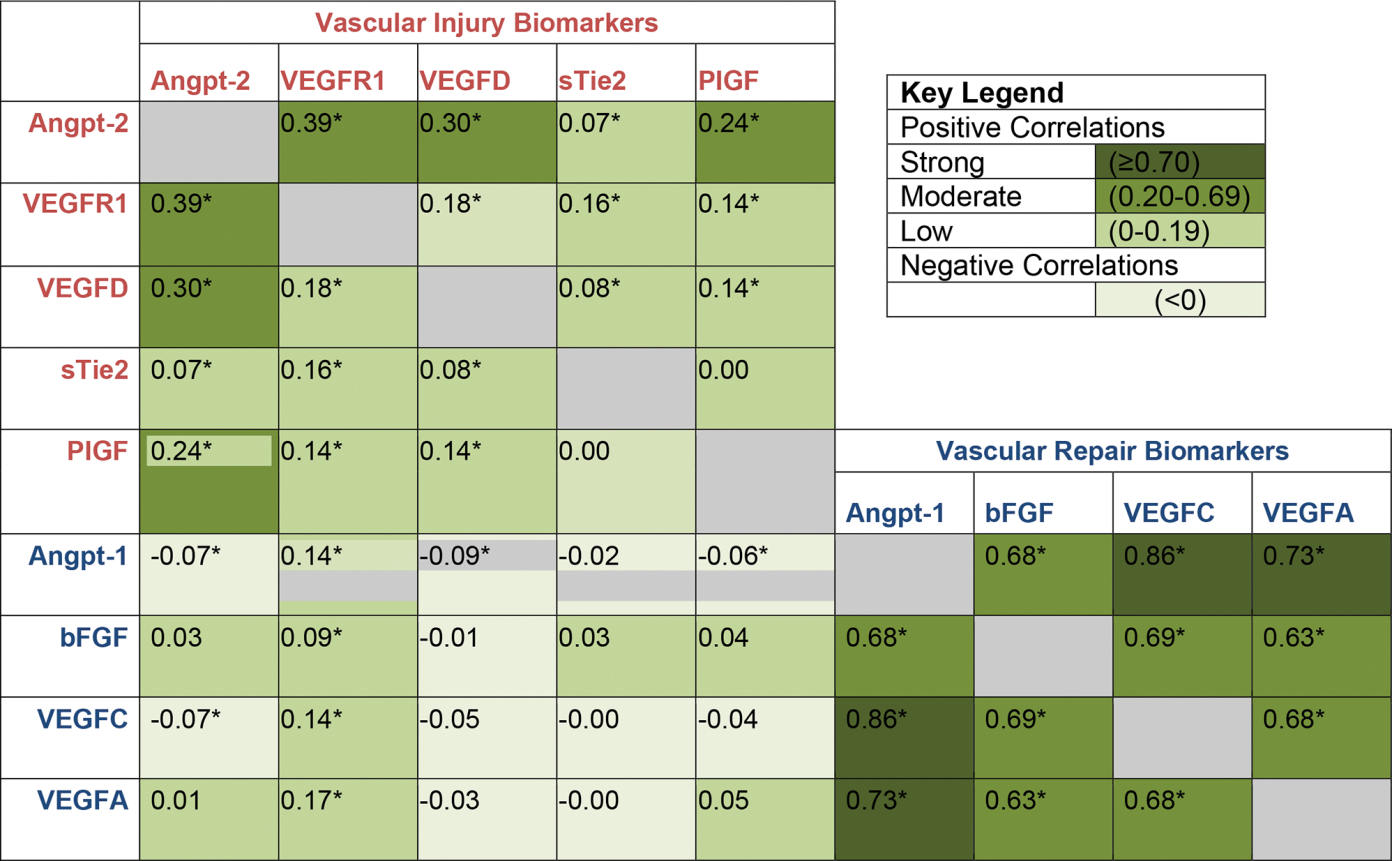
Biomarker correlations with heat map. *indicates statistically significant correlations (*P* < 0.05). Vascular repair biomarkers (VEGF-A, VEGF-C, angpt-1, and bFGF) are significantly and positively correlated with one another with Spearman correlations ranging from r^2^ = 0.63 to 0.86. Alternatively, vascular injury biomarkers (angpt-2, VEGF-D, VEGFR1, and PlGF) are mostly negatively correlated with vascular repair biomarkers

Vascular repair biomarkers (VEGF-A, VEGF-C, angpt-1, and bFGF) were significantly and positively correlated with one another. Spearman correlations ranged from *r* = 0.63 to 0.86, shown in Fig. 1. By contrast, injury biomarkers (angpt-2, VEGF-D, VEGFR1, and PlGF) were modestly and negatively correlated with the repair biomarker, angpt-1. The functions of each of the nine biomarkers can be found in Supplementary Table 3. Furthermore, biomarkers concentrations differed significantly among clusters (Supplementary Table 4). Higher concentrations of injury biomarkers (i.e. angpt-2, PlGF, sTie2, VEGFD, and VEGFR1) were shown among patients in the Injury Phenotype (Cluster 1), relative to Cluster 2 and Cluster 3. Conversely, higher relative concentrations of repair biomarkers: angpt-1, VEGFA, VEGFC and bFGF were seen in the Repair Phenotype (Cluster 2). Furthermore, the Dormant Phenotype displayed the lowest average concentrations of both repair and injury biomarkers.

**Fig. 2:**
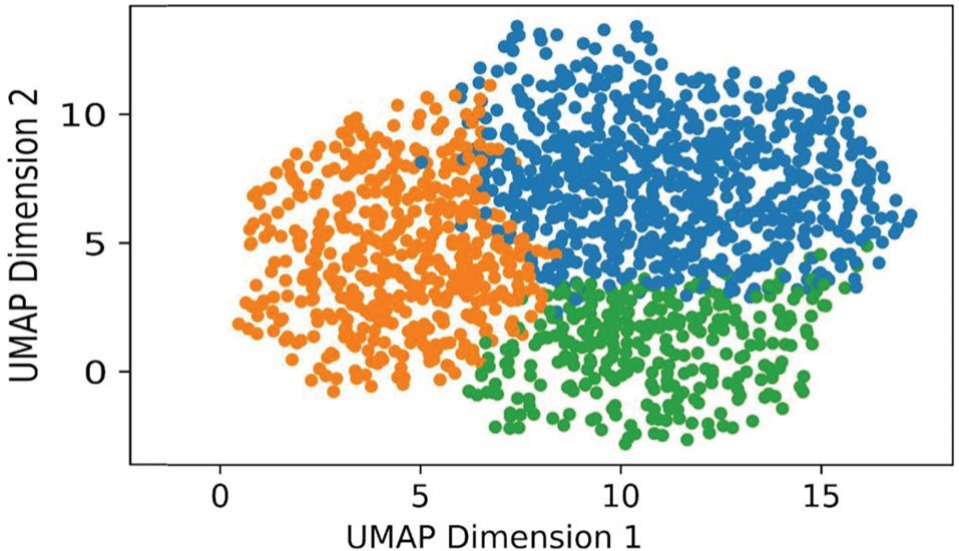
Differentiation between three biomarker-derived phenotypes. For visualization, the log-transformed and robust standardized biomarkers are projected to 2 dimensions using the UMAP algorithm. The labels given by the Spectral Clustering algorithm are used to color-code the data points. Green represents the Vascular Injury Phenotype (Cluster 1). Blue represents the Vascular Repair Phenotype (Cluster 2). Orange represents the Dormant Phenotype (Cluster 3). List of biomarkers used: Angiopoietin (angpt)-1, angpt-2, vascular endothelial growth factor (VEGF)-A, VEGF-C, VEGF-d, VEGF receptor 1 (R1), solubleTie-2 (sTie-2), placental growth factor (PlGF), and b-fibroblast growth factor (bFGF)

There was good differentiation between the biomarker-derived clusters, as shown in Fig. 2.

### Baseline characteristics of patients

This study included 1497 hospitalized patients, half of whom (746) were diagnosed with AKI during their hospitalization. Cluster analysis stratified 302 patients in Cluster 1 (Injury Phenotype), 728 patients in Cluster 2 (Repair Phenotype), and 467 patients in Cluster 3 (Dormant Phenotype). The median age was 66 years, with an interquartile range (IQR) of (57, 74). 196 patients (13%) were Black, and 555 (37%) patients were female, as shown in Table 1. Significantly higher rates of baseline HF, lung disease, CVD, hypertension, AKI, CKD, and diabetes were present among patients with the Injury Phenotype.

**Table 1:** Baseline and demographic characteristics stratified by Biomarker-derived phenotype

	Entire cohort *N* = 1497	Vascular injury phenotype (Cluster 1) *N* = 302	Vascular repair phenotype (Cluster 2) *N* = 728	Dormant phenotype (Cluster 3) *N* = 467	*P* value
**Demographics**					
Age, y	66 (57, 74)	67 (58, 74)	66 (56, 73)	65 (55, 75)	0.284
Female, n (%)	555 (37%)	104 (34%)	300 (41%)	149 (32%)	0.009
Black, n (%)	196 (13%)	39 (13%)	93 (13%)	64 (14%)	0.770
**Baseline Comorbidities**					
Ever Smoker, n (%)	867 (58%)	188 (62%)	379 (52%)	300 (64%)	< 0.001
Alcohol Use, n (%)	705 (47%)	122 (41%)	329 (45%)	251 (54%)	0.003
BMI, kg/m^2^	30 (26, 35)	29 (25, 36)	30 (26, 35)	29 (26, 34)	0.561
HF, n (%)	319 (21%)	127 (42%)	133 (18%)	57 (12%)	< 0.001
CLD, n (%)	59 (4%)	26 (9%)	15 (2%)	18 (4%)	< 0.001
COPD, n (%)	329 (22%)	86 (28%)	157 (22%)	84 (18%)	0.026
CVD, n (%)	680 (45%)	156 (52%)	290 (40%)	230 (49%)	0.001
Hypertension, n (%)	1118 (74%)	242 (80%)	542 (74%)	331 (70%)	0.029
ICU, n (%)	996 (66%)	211 (70%)	354 (49%)	425 (91%)	< 0.001
eGFR mL/min/1.73m^2^	67 (51, 87)	57 (40, 80)	69 (52, 88)	73 (55, 90)	< 0.001
Creatinine mg/dL	1.06 (0.87, 1.31)	1.2 (0.98, 1.6)	1.03 (0.84, 1.27)	1.02 (0.85, 1.23)	< 0.001
Diabetes, n (%)	642 (43%)	163(54%)	289 (40%)	187 (40%)	< 0.001
Sepsis, n (%)	143 (10%)	34 (11%)	61 (8%)	47 (10%)	0.444
CKD, n (%)	594 (40%)	163 (54%)	281 (39%)	147 (31%)	< 0.001
AKI during index hospitalization, n (%)	746 (50%)	181 (60%)	349 (48%)	212 (45%)	< 0.001
**Lab measurements at time of biomarker measurements (3 months after hospitalization)**					
BUN mg/dL	20 (15, 26)	24 (18, 36)	19 (15, 25)	19 (15, 25)	< 0.001
Creatinine mg/dL	1.03 (0.84, 1.33)	1.23 (0.97, 1.61)	1.01 (0.83, 1.24)	0.99 (0.83, 1.26)	< 0.001
eGFR mL/min/1.73m^2^	68.2 (50.2, 89.1)	54.4 (39.8, 74.1)	70.6 (52.7, 89.9)	72.6 (54.6, 92.3)	< 0.001
Cystatin C, mg/L	1.3 (1.0, 1.7)	1.7 (1.3, 2.2)	1.3 (1, 1.7)	1.3 (1, 1.6)	< 0.001
HDL, mg/dL	45 (37, 56)	43 (35, 54)	45 (38, 55)	46 (39, 57)	0.031
LDL, mg/dL	87 (67, 114)	76 (63, 104)	91 (72, 119)	86 (63, 114)	< 0.001
Potassium, meq/L	4.4 (4.1, 4.7)	4.4 (4, 4.7)	4.4 (4.1, 4.7)	4.4 (4.1, 4.6)	0.514
PTH, ng/L	50 (37, 71)	62 (42, 91)	48 (36, 66)	49 (35, 67)	< 0.001
Phosphorus, mg/dL	3.5 (3.2, 3.9)	3.7 (3.3, 4.1)	3.5 (3.2, 4.0)	3.5 (3.2, 3.8)	0.005
CRP, ug/mL	3.56 (1.51, 7.63)	6 (3, 12)	3 (1, 7)	3 (1, 6)	< 0.001
NT-proBNP	290 (88, 902)	1370 (477, 3006)	189 (65, 507)	254 (80, 637)	< 0.001
Troponin	16.9 (10.4, 28.7)	28.1 (16.8, 46.6)	14.8 (9.4, 25.7)	15.5 (9.7, 23.1)	< 0.001

Data are presented at median (IQR) or numbers (%)

AKI, acute kidney injury; BMI, body mass index; BUN, blood urea nitrogen; CKD, chronic kidney disease; CLD, chronic lung disease; COPD, chronic obstructive pulmonary disease; CRP, c-reactive protein; CVD, cardiovascular disease; eGFR, estimated glomerular filtration rate; HDL, high-density lipoprotein; HF, heart failure; ICU, intensive care unit; LDL, low-density lipoprotein; NT-proBNP, N-terminal-pro hormone b-type natriuretic peptide; PTH, parathyroid hormone

List of biomarkers used to derive cluster phenotypes: Angiopoietin (angpt)-1, angpt-2, vascular endothelial growth factor (VEGF)-A, VEGF-C, VEGF-d, VEGF receptor 1 (R1), solubleTie-2 (sTie-2), placental growth factor (PlGF), and b-fibroblast growth factor (bFGF)

In multinomial regression analysis, factors significantly associated with membership in the Injury Phenotype (as opposed to Repair) included: baseline HF, chronic lung disease, diabetes, being in the ICU or on diuretics during hospitalization, and smoker status prior to index hospitalization (Table 2). On the other hand, the presence of co-morbidities such as HF, hypertension, CKD, and AKI at time of index hospitalization decreased the likelihood of Dormant Phenotype membership, compared to Repair Phenotype. This stands in contrast with the association between admission to the ICU and likelihood of membership in the Dormant Phenotype.

**Table 2:** Multinomial logistic regression analysis of factors associated with Biomarker-derived phenotype membership

Factors	Vascular Injury Phenotype vs. Vascular Repair Phenotype (Cluster 1 vs. 2) aOR (95% CI)	Dormant Phenotype vs. Vascular Repair Phenotype (Cluster 3 vs. 2) aOR (95% CI)
Baseline variables		
Age	1.01 (0.99–1.03); *P* = 0.239	1.00 (0.99–1.02); *P* = 0.556
Female	0.99 (0.69–1.42); *P* = 0.999	0.97 (0.70–1.34); *P* = 0.846
Black	0.96 (0.73–1.26); *P* = 0.289	0.67 (0.51–0.87); *P* = 0.003
Smoker	1.71 (1.01–2.89); *P* = 0.026	1.64 (1.04–2.57); *P* = 0.040
BMI	1.01 (0.99–1.04); *P* = 0.230	1.01 (0.99–1.03); *P* = 0.363
HF	2.15 (1.50–3.09); *P* < 0.001	0.51 (0.34–0.77); *P* = 0.006
CLD	5.88 (2.73–12.63); *P* < 0.001	2.34 (1.03–5.30); *P* = 0.126
COPD	1.14 (0.80–1.63); *P* = 0.667	0.77 (0.54–1.09); *P* = 0.334
CVD	0.86 (0.61–1.22); *P* = 0.657	1.11 (0.81–1.51); *P* = 0.815
Hypertension	0.74 (0.49–1.11); *P* = 0.184	0.61 (0.43–0.86); *P* = 0.020
ICU	1.60 (1.03–2.48); *P* < 0.001	12.48 (8.43–18.48); *P* < 0.001
Diabetes	1.47 (1.05–2.06); *P* = 0.024	1.17 (0.87–1.58); *P* = 0.296
Sepsis	1.11 (0.64–1.93); *P* = 0.303	1.07 (0.67–1.72); *P* = 0.778
CKD	0.81 (0.47–1.39)); *P* = 0.349	0.57 (0.36–0.92); *P* = 0.020
Variables at hospitalization		
AKI	0.98 (0.70–1.37)); *P* = 0.709	0.67 (0.50–0.90); *P* = 0.008
eGFR	0.99 (0.98–1.01)); *P* = 0.259	0.99 (0.98–1.00); *P* = 0.143
Vasopressor use	1.21 (0.82–1.78)); *P* = 0.972	0.74 (0.54–1.02); *P* = 0.182
ACE inhibitor use	1.11 (0.77–1.60)); *P* = 0.434	1.16 (0.83–1.61); *P* = 0.674
ARB use	0.75 (0.44–1.27)); *P* = 0.565	0.84 (0.11–6.39); *P* = 0.436
Diuretic use	2.29 (1.57–3.33)); *P* < 0.001	0.86 (0.62–1.17); *P* = 0.334
NSAID use	1.26 (0.87–1.84)); *P* = 0.047	1.59 (1.19–2.13); *P* = 0.002
3-month variable		
3-month Creatinine	1.84 (1.21–2.81)); *P* = 0.001	1.08 (0.63–1.85); *P* = 0.773

AKI, acute kidney injury; BMI, body mass index; BUN, blood urea nitrogen; CKD, chronic kidney disease; CLD, chronic lung disease; COPD, chronic obstructive pulmonary disease; CRP, c-reactive protein; CVD, cardiovascular disease; eGFR, estimated glomerular filtration rate; HDL, high-density lipoprotein; HF, heart failure; ICU, intensive care unit; LDL, low-density lipoprotein; PTH, parathyroid hormone

List of biomarkers used to derive cluster phenotypes: Angiopoietin (angpt)-1, angpt-2, vascular endothelial growth factor (VEGF)-A, VEGF-C, VEGF-d, VEGF receptor 1 (R1), solubleTie-2 (sTie-2), placental growth factor (PlGF), and b-fibroblast growth factor (bFGF)

### Associations between biomarker-derived clusters and the primary outcome of HF

**Fig. 3:**
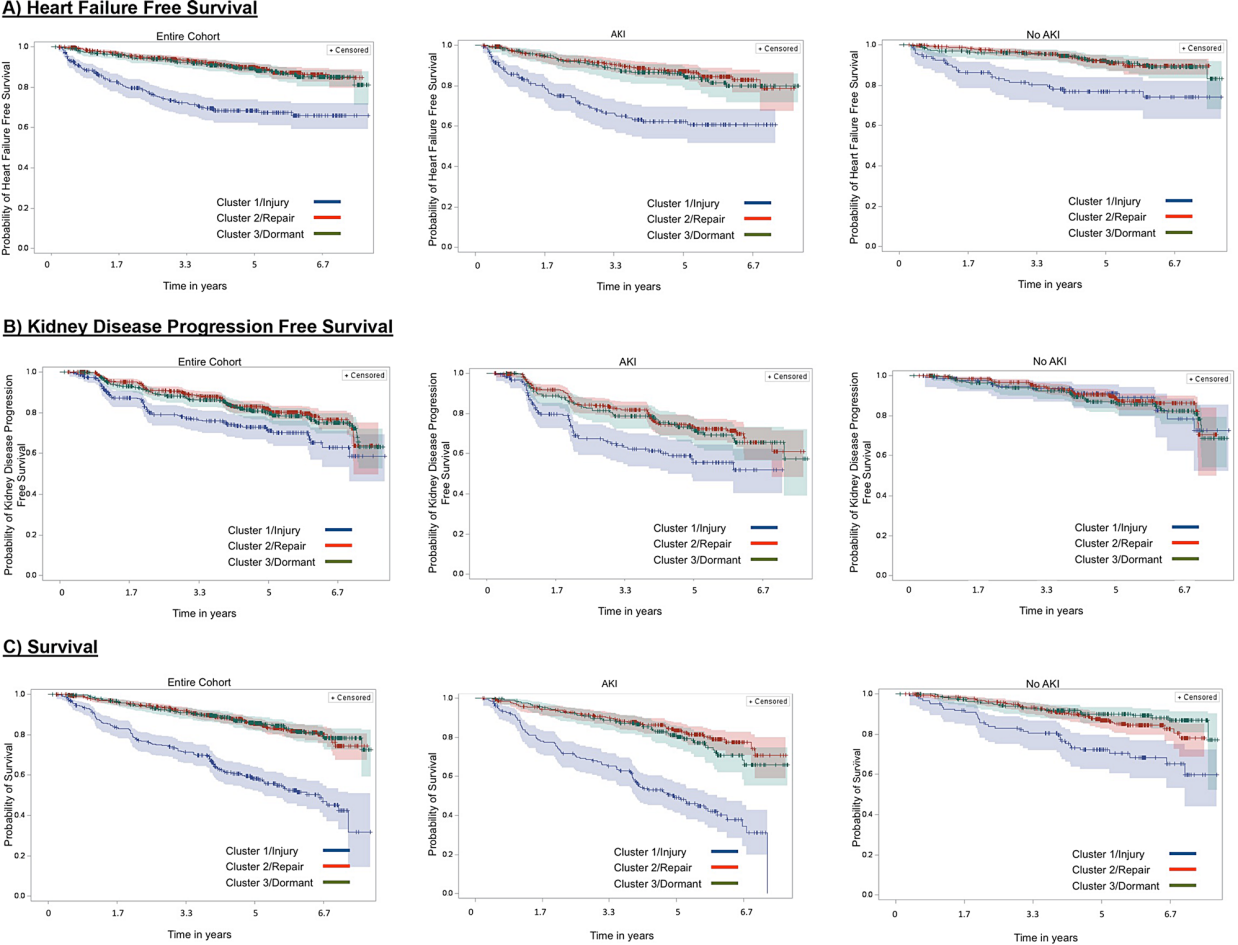
Kaplan Meier curves for the probabilities of heart failure event-free survival, kidney disease progression-free survival, and overall survival, overall and stratified by initial AKI status. The blue line represents participants with the Vascular Injury Phenotype (Cluster 1, n=302). The red line represents participants with the Vascular Repair Phenotype (Cluster 2, n=728). The green line represents participants with the Dormant Phenotype (Cluster 3, n=467). All inference testing for the above curves were significant with P-values <.001 except for the probability of kidney disease progression free survival among those without AKI (p=0.81)x0025

The median time to a HF event was 1.83 years (IQR: 0.79–3.48). Among patients in the Injury Phenotype cluster, 28% experienced a HF event, compared to only 12% and 10% of those in the Dormant and Repair Phenotype clusters, respectively (Table 3; Fig. 3a). In unadjusted analysis, patients within the Injury Phenotype cluster were 3.59 times more likely to experience a HF event [HR 3.59 (95% CI: 2.62–4.92)] as compared to those in the Repair Phenotype cluster (Table 4). The Dormant Phenotype cluster was not significantly associated with HF in unadjusted analysis.

**Table 3:** Differences in frequency of events per biomarker-derived phenotype

Event	Vascular Injury Phenotype	Vascular Repair Phenotype	Dormant Phenotype	*P* value
**Entire Cohort**				
	**(n = 302)**	**(n = 728)**	**(n = 467)**	
Heart failure	84 (28%)	72 (10%)	54 (12%)	< 0.001
Kidney disease progression	74 (25%)	124 (17%)	95 (20%)	0.020
All-cause mortality	130 (43%)	108 (15%)	74 (16%)	< 0.001
**AKI**				
	**(n = 181)**	**(n = 349)**	**(n = 212)**	
Heart failure	58 (32%)	44 (13%)	32 (15%)	< 0.001
Kidney disease progression	60 (33%)	84 (24%)	57 (27%)	0.083
All-cause mortality	95 (52%)	60 (18%)	47 (22%)	< 0.001
**Non-AKI**				
	**(n = 121)**	**(n = 379)**	**(n = 255)**	
Heart failure	26 (21%)	28 (7%)	22 (9%)	< 0.001
Kidney disease progression	14 (12%)	40 (11%)	38 (15%)	0.254
All-cause mortality	35 (29%)	48 (13%)	27 (11%)	< 0.001

Kidney disease progression is defined as “the combined outcome of CKD incidence, CKD progression, and end-stage renal disease or receipt of a kidney transplant.”

**Table 4:** Associations between biomarker-derived phenotypes and heart failure events

	Unadjusted HR (95% CI)	Adjusted aHR (95% CI)	Parsimonious model aHR (95% CI)
**Entire Cohort**			
Vascular Injury Phenotype	3.59 (2.62–4.92) *P* < 0.001	2.24 (1.57–3.19) *P* < 0.001	2.42 (1.71–3.42) *P* < 0.001
Dormant Phenotype	1.09 (0.76–1.55) *P* = 0.646	1.92 (1.19–3.11) *P* = 0.008	2.07 (1.30–3.31) *P* = 0.002
Vascular Repair Phenotype	(ref)	(ref)	(ref)
**Acute Kidney Injury**			
Vascular Injury Phenotype	3.42 (2.31–5.07) *P* < 0.001	2.12 (1.35–3.34) *P* = 0.001	2.30 (1.50–3.56) *P* < 0.001
Dormant Phenotype	1.16 (0.73–1.83) *P* = 0.533	1.48 (0.79–2.79) *P* = 0.224	1.70 (0.94–3.07) *P* = 0.082
Vascular Repair Phenotype	(ref)	(ref)	(ref)
**Non-Acute Kidney Injury**			
Vascular Injury Phenotype	3.42 (2.00–5.83) *P* < 0.001	2.94 (1.57–5.50) *P* = 0.001	3.31 (1.79–6.14) *P* < 0.001
Dormant Phenotype	1.04 (0.59–1.82) *P* = 0.902	2.40 (1.12–5.12) *P* = 0.024	2.30 (1.08–4.88) *P* = 0.030
Vascular Repair Phenotype	(ref)	(ref)	(ref)

Type 3 P-value was < 0.01 for the interaction term between AKI and the relationship between clusters and HF

**Adjusted**: Age, gender, race, ethnicity, body mass index, ever smoker, baseline heart failure, baseline chronic lung disease, baseline chronic obstructive pulmonary disease, baseline cardiovascular disease, baseline hypertension, baseline chronic kidney disease, baseline eGFR, baseline diabetes, admission to the intensive care unit during index hospitalization, diagnosis of sepsis at index hospitalization, vasopressor use during index hospitalization, angiotensin-converting enzyme inhibitor, angiotensin receptor blockers, diuretics, and non-steroidal anti-inflammatory drugs, center, serum creatinine at 3 month (to account for biomarker clearance), proteinuria at 3 month

**Parsimonious model**: Age, gender, race, ethnicity, baseline heart failure, baseline chronic obstructive pulmonary disease, baseline cardiovascular disease, baseline hypertension, baseline diabetes, baseline chronic kidney disease, baseline eGFR, admission to the intensive care unit during index hospitalization, diagnosis of sepsis at index hospitalization, center, serum creatinine at 3 months, proteinuria at 3 months

When adjusting for demographic variables, cardiac risk factors, and other variables as listed in Table 4, the Injury Phenotype remained significantly and independently associated with HF. Injury cluster patients exhibited 2.24 times the risk of experiencing a HF event [aHR 2.24 (95% CI: 1.57–3.19)] relative to Repair patients. Notably, after adjusting for confounding variables, the Dormant Phenotype cluster gained significance, with Dormant patients showing 1.92 times the risk of a HF event [aHR 1.92 (95%CI: 1.19–3.11)]. The Parsimonious Models showed similar results as shown in Table 4.

AKI status was found to be a significant effect modifier within the relationship between biomarker-derived clusters and HF, with an interaction *P*-value of < 0.01. The relationship between the Injury Phenotype cluster and HF remained significant among patients with AKI [aHR 2.12 (95%CI: 1.35–3.34)]; and among those without AKI [aHR 2.94 (95%CI: 1.57–5.50)]. Among Dormant Phenotype patients, only those without AKI continued to have a significant and independent relationship with the development of HF. By contrast, those with AKI lost significance (Table 4). In addition, when the biomarkers were stratified by AKI stages at index hospitalization, the distribution of three out of the nine biomarkers was significantly different (Supplementary Table 5).

Furthermore, we adjusted for NT-proBNP and troponin at the 3-month timepoint. We identified that the Injury Phenotype continued to be significantly associated with HF, relative to the Repair Phenotype [aHR 2.12 (95% CI: 1.46–3.07)], as well as the Dormant Phenotype with 67% increased risk of HF events [aHR 1.67 (95% CI: 1.02–2.74)] as shown in Table 5. Competing risk analysis revealed that the Injury Phenotype remained significantly associated with the composite outcome of HF or death [aHR 2.24 (95% CI:1.75–2.87), but the Dormant Phenotype was not statistically significant in multivariable analysis (Table 5).

**Table 5:** Associations between biomarker-derived phenotypes and heart failure events adjusted for pro-BNP and troponin

	Unadjusted HR (95% CI)	Adjusted aHR (95% CI)
**Entire Cohort**		
Vascular Injury Phenotype	3.59 (2.62–4.92), *P* < 0.001	2.11 (1.46–3.07), *P* < 0.001
Dormant Phenotype	1.09 (0.76–1.55), *P* = 0.646	1.67 (1.02–2.74), *P* = 0.042
Vascular Repair Phenotype	(ref)	(ref)
**Acute Kidney Injury**		
Vascular Injury Phenotype	3.42 (2.31–5.07), *P* < 0.001	2.04(1.26–3.31), *P* = 0.004
Dormant Phenotype	1.16 (0.73–1.83), *P* = 0.533	1.29 (0.67–2.49), *P* = 0.449
Vascular Repair Phenotype	(ref)	(ref)
**Non-Acute Kidney Injury**		
Vascular Injury Phenotype	3.42 (2.00–5.83), *P* < 0.001	2.27 (1.13–4.59), *P* = 0.022
Dormant Phenotype	1.04 (0.59–1.82), *P* = 0.902	2.46 (1.13–5.34), *P* = 0.023
Vascular Repair Phenotype	(ref)	(ref)

Type 3 P-value was < 0.01 for the interaction term between AKI and the relationship between clusters and HF.

**Adjusted**: age, gender, race, ethnicity, body mass index, ever smoker, baseline heart failure, baseline chronic lung disease, baseline chronic obstructive pulmonary disease, baseline cardiovascular disease, baseline hypertension, baseline chronic kidney disease, baseline eGFR, baseline diabetes, admission to the intensive care unit during index hospitalization, diagnosis of sepsis at index hospitalization, vasopressor use during index hospitalization, angiotensin-converting enzyme inhibitor, angiotensin receptor blockers, diuretics, and non-steroidal anti-inflammatory drugs, center, serum creatinine at 3 month (to account for biomarker clearance), proteinuria at 3 month + N-terminal-pro hormone b-type natriuretic peptide and troponin at 3 months

### Associations between biomarker-derived clusters and the secondary outcomes of kidney disease progression and all-cause mortality

The median time to the development of kidney disease progression was 2.08 years (IQR: 1.12–3.98). Among participants in the Injury Phenotype, 25% developed kidney disease progression, as compared to 20% and 17% of those in the Dormant and Repair Phenotypes, respectively (Table 3; Fig. 3b). Injury Phenotype patients had a 1.43 higher risk of kidney disease progression independent of traditional risk factors [aHR 1.43 (95% CI: 1.02–2.01)] (Supplementary Table 7). The Dormant Phenotype was also significantly and independently associated with kidney disease progression [aHR 1.60 (95%CI: 1.12–2.28)]. AKI significantly modified the relationship between clusters and kidney disease progression, with an interaction *P*-value of < 0.01. Among those with AKI, the relationships between both the Injury and Dormant Phenotypes and kidney disease progression were significant and independent of traditional risk factors. However, there were no significant associations among those without AKI (Supplementary Table 7).

The median time to death was 2.87 years (IQR: 1.43–4.26). 43% of Injury Phenotype patients died, relative to 16% and 15% of Dormant and Repair patients, respectively (Table 3; Fig. 3c). Injury patients were 2.6 times more likely to die as compared to Repair patients, independent of traditional risk factors (Supplementary Table 8). By contrast, no significant association was found between the Dormant Phenotype and all-cause mortality. AKI status significantly modified the relationship between cluster and all-cause mortality, with an interaction *P*-value of < 0.01. The relationship between the Injury Phenotype and all-cause mortality remained significant in patients with and without AKI. However, the association was stronger among AKI patients [aHR 3.04 (95%CI: 2.12–4.35)] vs. in those without AKI [aHR 2.02 (95%CI: 1.20–3.41)].

### Vascular biomarkers and prediction of future HF events or death at 3 years (Fig. 4)

**Fig. 4:**
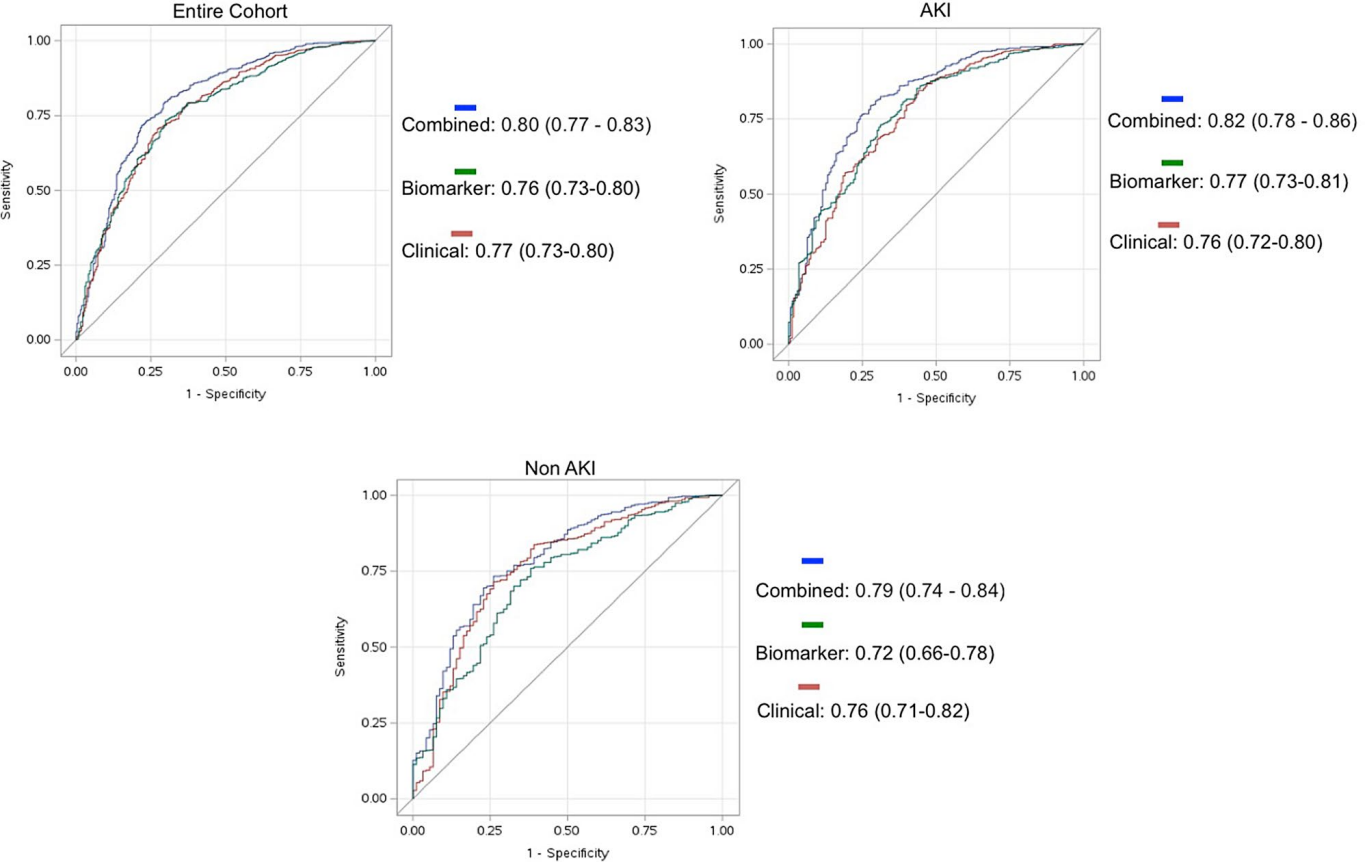
ROC curves for the prediction of heart failure events or death at 3 years using different models. Biomarker panel includes: Angiopoietin-1, angpt-2, vascular endothelial growth factor (VEGF)-A, VEGF-C, VEGF-d, VEGF receptor 1 (R1), solubleTie-2 (sTie-2), placental growth factor (PlGF), and b-fibroblast growth factor (bFGF). Clinical panel includes: demographics (age, gender, race, ethnicity); CKD and CHF at baseline; baseline eGFR; BMI, serum creatinine, and urine protein at 3 months; smoker status; COPD; CVD; sepsis; diabetes; ICU status; and admission center

Across the entire cohort, the areas under the ROC curves for all 9 vascular biomarkers, the LASSO-selected clinical model, and the combined biomarker and clinical model were 0.76 (95% CI: 0.73–0.80), 0.77 (95% CI: 0.73–0.80), and 0.80 (95% CI: 0.77–0.83), respectively. Among individuals who experienced HF or death at 3 years, 136 were correctly reclassified into having a higher risk and 129 were incorrectly reclassified into having a lower risk of the event, yielding an NRI for events of 0.026. Among those who did not experience HF or death at 3 years, 901 were correctly reclassified into having a lower risk while 335 were incorrectly reclassified into having a higher risk, resulting in an NRI for non-events of 0.458. The overall NRI for the addition of the biomarkers to the clinical model was 0.4845, indicating that the new model substantially improved risk classification, especially among non-events.

For participants with AKI, the areas under the ROC curvesg for all 9 vascular biomarkers, the LASSO-selected clinical model, and the combined biomarker and clinical model were 0.77 (95% CI: 0.73–0.81), 0.76 (95% CI: 0.72–0.80), and 0.82 (95% CI: 0.78–0.86), respectively. The change in AUC by the addition of the biomarkers to the clinical model was statistically significant (*P* < 0.001).

For non-AKI participants, the areas under the ROC curves for the biomarkers, the LASSO-selected clinical model, and combined model were 0.72 (95% CI: 0.66–0.78), 0.76 (95% CI: 0.71–0.82), and 0.79 (95% CI: 0.74–0.84), respectively. The change in AUC by adding the biomarker model to the clinical model was not statistically significant (*P* = 0.083).

## Discussion

In this ancillary study from ASSESS-AKI, we studied hospitalized patients with and without AKI. Blood samples were analyzed for 9 vascular biomarkers, measured 3 months post-discharge. Using an unsupervised cluster analysis, we found that these biomarkers could identify three clusters of patients— comprising an Injury, Repair, and Dormant Phenotype. These phenotypes were capable of differentiating participants at high risk of developing poor long-term outcomes. Relative to the Repair Phenotype, the Injury and Dormant Phenotypes were strong predictors of patients at increased risk of experiencing a subsequent HF event.

As for secondary outcomes, the Injury Phenotype was only associated with all-cause mortality; whereas the Dormant Phenotype was associated solely with kidney disease progression. The Injury Phenotype significantly and independently predicted hospitalization for HF in patients both with and without AKI, with a stronger association in non-AKI patients. The Dormant Phenotype was not significantly associated with HF among AKI patients. Furthermore, adding vascular biomarkers to clinical variables significantly improved prediction of HF or death at 3 years in the overall cohort and among AKI patients.

Notably, our analysis identified a group of patients capable of mounting a repair response 3 months post-discharge who had a lower risk of poor outcomes, relative to those with chronically elevated vascular injury markers. Furthermore, we found that Dormant Phenotype participants had a higher risk of poor outcomes, compared to Repair patients. This suggests that both Injury and Dormant Phenotype patients may be at higher risk of poor outcomes post-hospitalization. In fact, it is possible that the dormant group—characterized by low levels of both vascular injury and repair biomarkers—may represent individuals with an impaired biological responsiveness to injury. In this group, clinical injury during hospitalization may have failed to elicit a detectable injury response, leading to an absence of a subsequent repair process. This lack of adaptive response could explain why these patients had a higher risk of adverse outcomes, as opposed to those who demonstrated a measurable repair response. Nonetheless, due to the constraints of the study design, it is unclear if the participants’ vascular phenotypes, as defined by the 3-month biomarker measurements, are a consequence of the index hospitalization; or whether they pre-dated the index hospitalization (i.e. participants already exhibited a pre-existing vascular phenotype, which placed them at risk for hospitalization and poor outcomes).

It bears noting that we measured biomarkers at a 3-month outpatient post-discharge visit in order to best assess vascular repair processes after the initial injury. Given that acute hemodynamic and inflammatory changes occur in the early post-hospitalization period, we felt that measuring biomarkers at 3 months would provide better insight into longer-term vascular recovery and remodeling. Additionally, this was the earliest available time point after the index hospitalization, allowing us to capture post-injury physiological adaptations without the confounding influence of acute illness. Furthermore, because the secondary outcome of CKD was also measured at the three-month timepoint, we included AKI as an effect modifier in the relationship between clusters and kidney disease progression. Our analysis identified that AKI significantly modifies the association between biomarkers and kidney disease progression, with these biomarkers being predictive of CKD progression only in the setting of AKI. This suggests that the initial AKI episode likely influenced biomarker levels and contributed to their relationship with CKD progression.

Given that baseline or pre-AKI biomarker measurements were unavailable, it is possible that the biomarker concentrations observed 3-months after discharge measured those more vulnerable to having AKI. Our unadjusted analysis between the Dormant Phenotype and the outcome of HF was not statistically significant, but after adjustment for multiple confounders became significant. We attribute this change to confounding, as we likely did not account for influential variables in univariate analysis. When we included these confounders as covariates, the effect of Dormant Phenotype on HF become more apparent.

In ROC analysis, we accounted for participants that could have died prior to developing HF by using the composite outcome of HF or death at 3 years. The addition of the vascular biomarkers to routine clinical variables significantly improved prediction and reclassification among all hospitalized patients. Indeed, among AKI patients, biomarkers were stronger predictors of the composite outcome than the clinical variables.

These findings may be used to phenotype AKI patients [32] post-discharge to tailor cardio-protective therapy in high-risk groups– i.e. by use of SGLT2i, now considered a foundational therapy with both kidney and cardiovascular benefits [33, 34]. Additionally, our analyses show vascular markers to be significant not only for patients with AKI, but also for non-AKI patients. Non-AKI patients exhibited up to 3 times the risk of experiencing a HF event if they belonged to the Injury Phenotype. These relationships held true even after controlling for traditional risk factors, including troponin and NT-proBNP.

To our knowledge, this is the first study to rely exclusively on vascular biomarkers to identify clusters of hospitalized patients, with and without AKI, in an unsupervised approach. Contrary to supervised techniques, clustering without knowledge of outcomes is an ideal way to phenotype patients without needing validation [35, 36]. Our analysis also uniquely allows the algorithm to independently develop phenotypes to broaden our knowledge about different vascular biomarker domains, surpassing conclusions possible from a single pathway analysis [37].

Our analysis is limited in its use of an arbitrary number of clusters. We initially searched for 4 clusters, but the algorithm partitioned over 99% of the subjects into one of 3 clusters. Ultimately, we felt that a small number of cluster strata might obscure cohort heterogeneity; and a large number would limit generalizability. It is also important to note that the categorical terms “Injury”, “Repair” and “Dormant” were chosen for analytical ease. This terminology may not reflect the precise biology of the implicated biomarkers [38, 39]. Furthermore, future work incorporating longitudinal biomarker measurements might explore whether patients shift between clusters over time and how such transitions relate to clinical outcomes. Lastly, the use of more objective echocardiographic parameters to further characterize the type or severity of heart failure would have been valuable. However, these data were not available in this cohort. Future research should explore the role of echocardiographic markers in conjunction with vascular biomarkers to better characterize heart failure phenotypes.

In conclusion, our biomarker-derived unsupervised cluster analysis identified three distinct phenotypes: Injury, Repair, and Dormant. Hospitalized patients with an Injury or Dormant profile at 3 months post-discharge had a higher risk of HF events, with a significant association persisting between the Injury Phenotype and HF among patients with and without AKI. Superimposing the vascular biomarker panel upon pre-existing clinical variables significantly improved prediction of 3-year HF events or death. As the 9 biomarkers used in this analysis can be presently measured using fast and commercially available assays, it is our hope that such an analysis facilitates future bedside applications, affording an improved time-horizon to initiate guideline-directed therapy [34].

## Electronic supplementary material

Below is the link to the electronic supplementary material.

Supplementary Material 1

## Data Availability

The deidentified datasets used and/or analysed during the current study are available from the corresponding author on reasonable request.

## Abbreviations

AKI: Acute kidney injury
Angpt: Angiopoietin
bFGF: Basic fibroblast growth factor
CKD: Chronic kidney disease
eGFR: Estimated glomerular filtration rate
HF: Heart failure
PlGF: Placental growth factor
sTie2: Soluble Tie-2 receptor
VEGF: Vascular endothelial growth factor
VEGFR1: Vascular endothelial growth factor receptor 1
