# Supplementary material for: Using vascular biomarkers to assess heart failure event risk in hospitalized patients with and without AKI
Source: BMC Nephrol. 2025 Jun 2;26:271. doi: 10.1186/s12882-025-04169-1 (PMC12131712; doi:10.1186/s12882-025-04169-1)
Supplement: Supplementary file 1 — Supplementary Material 1 [file 12882_2025_4169_MOESM1_ESM.docx]

**SUPPLEMENTARY FILES:**

**SUPPLEMENTARY TABLE 1: Inter-assay coefficient of variance for the 9 vascular health biomarkers**

|  | |
| --- | --- |
| Biomarker | % Inter-assay coefficient of variance range  (mean, median) |
| Vascular Repair Biomarkers | |
| Angpt-1 | 0.0 – 42.1  (6.3, 4.8) |
| VEGFA | 0.0 – 11.5  (2.9, 2.4) |
| VEGFC | 0.0 – 32.8  (7.5, 5.7) |
| bFGF | 0.1 – 17.6  (4.7, 4.1) |
| Vascular Injury Biomarkers | |
| Angpt-2 | 0.0 – 12.4  (3.2, 2.5) |
| VEGFR1 | 0.0 – 12.1  (3.5, 2.9) |
| PGIF | 0.0 – 19.8  (3.9, 2.7) |
| Tie-2 | 0.1 – 20.5  (5.2, 3.9) |
| VEGFD | 0.0 – 18.9  (6.3, 5.7) |

Values obtained by repeating the same samples in singlet on a different day. Repeated samples were plated on the same day and underwent the same number of freeze-thaw cycles

**SUPPLEMENTARY TABLE 2: Range of detection of each biomarker using the MesoScale technology**

| Biomarker | Level | Range of detection |
| --- | --- | --- |
| Vascular Repair Biomarkers (pg/mL) | | |
| Angpt-1 | Upper limit | 8 x 10^5^ |
|  | Lower limit | 122.4 |
| VEGFA | Upper limit | 4900 |
|  | Lower limit | 1.352 |
| VEGFC | Upper limit | 49200 |
|  | Lower limit | 15.32 |
| bFGF | Upper limit | 4980 |
|  | Lower limit | 0.374 |
| Vascular Injury Biomarkers (pg/mL) | | |
| Angpt-2 | Upper limit | 4 x 10^5^ |
|  | Lower limit | 8.2 |
| VEGFR1 | Upper limit | 19660 |
|  | Lower limit | 2.34 |
| PGIF | Upper limit | 2420 |
|  | Lower limit | 0.45 |
| Tie-2 | Upper limit | 184000 |
|  | Lower limit | 71 |
| VEGFD | Upper limit | 56800 |
|  | Lower limit | 16.82 |

Clinically reportable range. Accounts for the method dilution factor

**SUPPLEMENTARY TABLE 3: Summary of Vascular Biomarker Functions**

| **Biomarker** | **Function** | **Cited Literature** |
| --- | --- | --- |
| **Biomarkers of Vascular Repair** | | |
| Angpt-1 | - elevated levels of Angpt-1 are associated with decreased CKD progression, HF, and mortality among AKI patients (Mansour 2022) - in AKI setting: reduces renal interstitial fibrosis by decreasing myoblast activation (Singh 2016) | Mansour SG et al. Angiopoietins as Prognostic Markers for Future Kidney Disease and Heart Failure Events after Acute Kidney Injury. J Am Soc Nephrol. 2022; 33(3):613-627.  Singh S et al. Tubular Overexpression of Angiopoietin-1 Attenuates Renal Fibrosis. PLoS One. 2016;11(7):e0158908. 2016 Jul 25. |
| VEGFA | - released by platelets upon endothelial injury; also released by macrophages during wound healing (Barrientos 2008) - prolongs vascular survival by inhibiting endothelial apoptosis (Ferrara 2001) - increased levels seen in patients with acute myocardial infarction or refractory angina (Lee 2004) | Barrientos S, Stojadinovic O, Golinko MS, Brem H, Tomic-Canic M. Growth factors and cytokines in wound healing. Wound Repair Regen. 2008;16(5):585-601.  Ferrara N, Gerber HP. The role of vascular endothelial growth factor in angiogenesis. Acta Haematol. 2001;106(4):148-156.  Lee KW, Lip GY, Blann AD. Plasma angiopoietin-1, angiopoietin-2, angiopoietin receptor tie-2, and vascular endothelial growth factor levels in acute coronary syndromes. Circulation. 2004;110(16):2355-2360. |
| VEGFC | - promotes angiogenesis in ischemic tissue (Witzenbichler 1998) - accelerates wound healing by promoting lymphangiogenesis (Brunner 2023) | Witzenbichler B, Asahara T, Murohara T, et al. Vascular endothelial growth factor-C (VEGF-C/VEGF-2) promotes angiogenesis in the setting of tissue ischemia. Am J Pathol. 1998;153(2):381-394.  Brunner LM, He Y, Cousin N, et al. Promotion of Lymphangiogenesis by Targeted Delivery of VEGF-C Improves Diabetic Wound Healing. Cells. 2023;12(3):472. |
| bFGF | - promotes angiogenesis by activating endothelial cell proliferation (Yamamoto 2020) | Yamamoto N, Oyaizu T, Enomoto M, et al. VEGF and bFGF induction by nitric oxide is associated with hyperbaric oxygen-induced angiogenesis and muscle regeneration. Sci Rep. 2020;10(1):2744. |
| **Biomarkers of Vascular Injury** | | |
| Angpt-2 | - increases vascular leakage, promotes inflammation, and exacerbates cardiac hypoxia - stimulates inflammation and arterial stiffness in the setting of CKD (Chang 2014) | Chang FC, Chiang WC, Tsai MH, et al. Angiopoietin-2-induced arterial stiffness in CKD. J Am Soc Nephrol. 2014;25(6):1198-1209. |
| VEGFR1 | - promotes pathological angiogenesis and inflammation (Ceci 2020) - excess VEGFR1 increases proteinuria and glomerular endothelial swelling (Tanabe 2020) | Ceci C, Atzori MG, Lacal PM, Graziani G. Role of VEGFs/VEGFR-1 Signaling and its Inhibition in Modulating Tumor Invasion: Experimental Evidence in Different Metastatic Cancer Models. Int J Mol Sci. 2020;21(4):1388. Published 2020 Feb 18.  Tanabe K, Wada J, Sato Y. Targeting angiogenesis and lymphangiogenesis in kidney disease. Nat Rev Nephrol. 2020;16(5):289-303. |
| PIGF | - stimulates vascular inflammation and plaque destabilization (Matsui 2015) - levels are significantly elevated in post-COVID and post-sepsis patients (Melhorn 2023) | Matsui M, Uemura S, Takeda Y, et al. Placental Growth Factor as a Predictor of Cardiovascular Events in Patients with CKD from the NARA-CKD Study. J Am Soc Nephrol. 2015;26(11):2871-2881.  Melhorn J, Alamoudi A, Mentzer AJ, et al. Persistence of inflammatory and vascular mediators 5 months after hospitalization with COVID-19 infection. Front Med (Lausanne). 2023;10:1056506 |
| Tie-2 | - main receptor for Angpt-1 signaling (Bilimoria 2019) - elevated levels seen in acute myocardial infarction and chronically inflamed patients (Lee 2004) | Bilimoria J, Singh H. The Angiopoietin ligands and Tie receptors: potential diagnostic biomarkers of vascular disease. J Recept Signal Transduct Res. 2019;39(3):187-193.  Lee KW, Lip GY, Blann AD. Plasma angiopoietin-1, angiopoietin-2, angiopoietin receptor tie-2, and vascular endothelial growth factor levels in acute coronary syndromes. Circulation. 2004;110(16):2355-2360. |
| VEGFD | - known endothelial cell mitogen (Achen 1998) - in the setting of coronary artery disease: elevated levels are significantly associated with all-cause death and cardiovascular death (Wada 2020) | Achen MG, Jeltsch M, Kukk E, et al. Vascular endothelial growth factor D (VEGF-D) is a ligand for the tyrosine kinases VEGF receptor 2 (Flk1) and VEGF receptor 3 (Flt4). Proc Natl Acad Sci U S A. 1998;95(2):548-553.  Wada H, Suzuki M, Matsuda M, et al. Distinct Characteristics of VEGF-D and VEGF-C to Predict Mortality in Patients With Suspected or Known Coronary Artery Disease. J Am Heart Assoc. 2020;9(9):e015761. |

**SUPPLEMENTARY TABLE 4: Differences in biomarker levels between clusters**

| **Biomarker** | **Vascular Injury Phenotype (n=302)** | **Vascular Repair Phenotype (n=728)** | **Dormant Phenotype (n=467)** | ***P* value** |
| --- | --- | --- | --- | --- |
| Vascular Repair Biomarkers (pg/mL) | | | | |
| Angpt-1 | 4424 (2289, 7500) | 8235 (5432, 12233) | 1767 (969, 2940) | <.001 |
| VEGFA | 150 (84. 257) | 232 (155, 394) | 77 (59, 108) | <.001 |
| VEGFC | 117 (82, 174) | 189 (139, 273) | 63 (47, 83) | <.001 |
| bFGF | 70 (25, 144) | 129 (75, 210) | 13 (6, 28) | <.001 |
| Vascular Injury Biomarkers (pg/mL) | | | | |
| Angpt-2 | 5420 (3525, 8648) | 1960 (1503, 2602) | 1914 (1505, 2728) | <.001 |
| sTie2 | 6741 (5982, 7654) | 6187 (5399, 7026) | 6219 (5515, 7067) | <.001 |
| VEGFD | 995 (778, 1288) | 666 (519, 844) | 678 (543, 855) | <.001 |
| PlGF | 9.66 (7.92, 11.87) | 8.72 (7.38, 10.58) | 8.59 (6.92 , 10.45) | <.001 |
| VEGFR1 | 126 (101, 153) | 87 (74, 102) | 77 (67, 91) | <.001 |

Angpt-1, angiopoietin-1; Angpt-2, angiopoietin-2; bFGF, basic fibroblast growth factor; PlGF, place, vascular endothelial growth factor ntal growth factor; sTie2, soluble Tie 2 receptor; VEGFA, vascular endothelial growth factor A; VEGFC, vascular endothelial growth factor C; VEGFD, vascular endothelial growth factor D; VEGFR1, vascular endothelial growth factor receptor 1

**SUPPLEMENTARY TABLE 5: Distribution of biomarker levels by AKI severity**

| **3m Biomarkers** | **No AKI**  **(n=755)** | **Stage 1**  **(n=536)** | **Stage 2**  **(n=114)** | **Stage 3**  **(n=92)** | **P-value** |
| --- | --- | --- | --- | --- | --- |
| PIGF (mean +/-SD) | 9.37 ± 9.24 | 9.42 ± 2.93 | 10.71 ± 15.99 | 9.58 ± 3.31 | 0.42 |
| Tie2 | 6296.66 ± 1440.74 | 6513.61 ± 1579.57 | 6583.27 ± 1496.60 | 6504.19 ± 1669.69 | 0.03 |
| VEGFD | 827.34 ± 2064.10 | 831.79 ± 376.33 | 787.25 ± 339.39 | 788.81 ± 360.31 | 0.99 |
| sFlt1 | 93.54 ± 38.58 | 100.31 ± 47.27 | 98.49 ± 39.46 | 158.28 ± 476.98 | <0.01 |
| Angpt-2 | 2645.47 ± 2193.03 | 3537.08 ± 3435.06 | 3516.37 ± 2962.07 | 4915.90 ± 6936.54 | <0.01 |
| VEGFA | 221.00 ± 209.02 | 221.99 ± 239.17 | 268.43 ± 385.95 | 246.06 ± 283.53 | 0.21 |
| VEGFC | 159.41 ± 115.50 | 151.16 ± 107.62 | 146.82 ± 92.67 | 156.53 ± 110.56 | 0.48 |
| bFGF | 97.30 ± 100.75 | 100.99 ± 109.63 | 114.67 ± 171.62 | 122.24 ± 134.16 | 0.13 |
| Angpt-1 | 6691.50 ± 5885.95 | 6099.43 ± 5393.37 | 6075.47 ± 5164.24 | 6457.50 ± 5205.69 | 0.27 |

**SUPPLEMENTARY TABLE 6: Competing risk analysis for the association between biomarker-derived phenotypes and HF or death.**

|  | **Unadjusted**  HR (95% CI) | **Adjusted**  aHR (95% CI) |
| --- | --- | --- |
|  | **Entire Cohort** | |
| Vascular Injury Phenotype | 1.76 (1.32 - 2.35)  *P*<0.001 | 2.24 (1.75 - 2.87)  *P*<0.001 |
|  |  |  |
| Dormant Phenotype | 1.07 (0.82 - 1.40)  *P*=0.617 | 1.24 (0.89 - 1.72)  *P*=0.216 |
|  |  |  |
| Vascular Repair Phenotype | (ref) | (ref) |
|  | **Acute Kidney Injury** | |
| Vascular Injury Phenotype | 1.91 (1.37 - 2.67)  *P*<0.001 | 2.60 (1.89 - 3.56)  *P*<0.001 |
|  |  |  |
| Dormant Phenotype | 1.07 (0.76 - 1.49)  *P*=0.715 | 1.35 (0.87 - 2.08)  *P*=0.180 |
|  |  |  |
| Vascular Repair Phenotype | (ref) | (ref) |
|  | **Non-Acute Kidney Injury** | |
| Vascular Injury Phenotype | 1.09 (0.59 - 2.00)  *P*=0.785 | 1.98 (1.28 - 3.06)  *P*=0.002 |
|  |  |  |
| Dormant Phenotype | 1.17 (0.74 - 1.84)  *P*=0.495 | 1.03 (0.61 - 1.72)  *P*=0.916 |
|  |  |  |
| Vascular Repair Phenotype | (ref) | (ref) |

Type 3 P-value was <0.01 for the interaction term between AKI and the relationship between clusters and CKD progression.

**Adjusted**: age, gender, race, ethnicity, body mass index, ever smoker, baseline heart failure, baseline chronic lung disease, baseline chronic obstructive pulmonary disease, baseline cardiovascular disease, baseline hypertension, baseline chronic kidney disease, baseline eGFR, baseline diabetes, admission to the intensive care unit during index hospitalization, diagnosis of sepsis at index hospitalization, vasopressor use during index hospitalization, angiotensin-converting enzyme inhibitor, angiotensin receptor blockers, diuretics, and non-steroidal anti-inflammatory drugs, center, serum creatinine at 3 month (to account for biomarker clearance), proteinuria at 3 month

**Parsimonious Model:** age, gender, race, ethnicity, baseline heart failure, baseline chronic obstructive pulmonary disease, baseline cardiovascular disease, baseline hypertension, baseline diabetes, baseline chronic kidney disease, baseline eGFR, admission to the intensive care unit during index hospitalization, diagnosis of sepsis at index hospitalization, center, serum creatinine at 3 months, proteinuria at 3 months

**SUPPLEMENTARY TABLE 7: Association between biomarker-derived phenotypes and kidney disease progression**

|  | **Unadjusted**  HR (95% CI) | **Adjusted**  aHR (95% CI) | **Parsimonious Model**  aHR (95% CI) |
| --- | --- | --- | --- |
|  | **Entire Cohort** | | |
| Vascular Injury Phenotype | 1.76 (1.32 - 2.35)  *P*<0.001 | 1.43 (1.02 - 2.01)  *P*=0.040 | 1.39 (0.99 - 1.94)  *P*=0.055 |
|  |  |  |  |
| Dormant Phenotype | 1.07 (0.82 - 1.40)  *P*=0.617 | 1.60 (1.12 - 2.28)  *P*=0.010 | 1.60 (1.13 - 2.25)  *P*=0.008 |
|  |  |  |  |
| Vascular Repair Phenotype | (ref) | (ref) | (ref) |
|  | **Acute Kidney Injury** | | |
| Vascular Injury Phenotype | 1.91 (1.37 - 2.67)  *P*<0.001 | 1.51 (1.02 - 2.23)  *P*=0.038 | 1.38 (0.94 - 2.02)  *P*=0.098 |
|  |  |  |  |
| Dormant Phenotype | 1.07 (0.76 - 1.49)  *P*=0.715 | 1.84 (1.16 - 2.93)  *P*=0.010 | 1.80 (1.15 - 2.82)  *P*=0.010 |
|  |  |  |  |
| Vascular Repair Phenotype | (ref) | (ref) | (ref) |
|  | **Non-Acute Kidney Injury** | | |
| Vascular Injury Phenotype | 1.09 (0.59 - 2.00)  *P*=0.785 | 1.26 (0.58 - 2.76)  *P*=0.556 | 1.09 (0.51 - 2.32)  *P*=0.832 |
|  |  |  |  |
| Dormant Phenotype | 1.17 (0.74 - 1.84)  *P*=0.495 | 1.29 (0.67 - 2.47)  *P*=0.470 | 1.34 (0.72 - 2.48)  *P*=0.357 |
|  |  |  |  |
| Vascular Repair Phenotype | (ref) | (ref) | (ref) |

Type 3 P-value was <0.01 for the interaction term between AKI and the relationship between clusters and CKD progression.

**Adjusted**: age, gender, race, ethnicity, body mass index, ever smoker, baseline heart failure, baseline chronic lung disease, baseline chronic obstructive pulmonary disease, baseline cardiovascular disease, baseline hypertension, baseline chronic kidney disease, baseline eGFR, baseline diabetes, admission to the intensive care unit during index hospitalization, diagnosis of sepsis at index hospitalization, vasopressor use during index hospitalization, angiotensin-converting enzyme inhibitor, angiotensin receptor blockers, diuretics, and non-steroidal anti-inflammatory drugs, center, serum creatinine at 3 month (to account for biomarker clearance), proteinuria at 3 month

**Parsimonious Model:** age, gender, race, ethnicity, baseline heart failure, baseline chronic obstructive pulmonary disease, baseline cardiovascular disease, baseline hypertension, baseline diabetes, baseline chronic kidney disease, baseline eGFR, admission to the intensive care unit during index hospitalization, diagnosis of sepsis at index hospitalization, center, serum creatinine at 3 months, proteinuria at 3 months

**SUPPLEMENTARY TABLE 8: Association between biomarker-derived phenotypes and all-cause mortality**

|  | **Unadjusted**  HR (95% CI) | **Adjusted**  aHR (95% CI) | **Parsimonious Model**  aHR (95% CI) |
| --- | --- | --- | --- |
| **Entire Cohort** | | | |
| Vascular Injury Phenotype | 3.40 (2.63 - 4.39)  *P*<0.001 | 2.58 (1.93 - 3.43)  *P*<0.001 | 2.67 (2.01 - 3.53)  *P*<0.001 |
|  |  |  |  |
| Dormant Phenotype | 0.96 (0.71 - 1.30)  *P*=0.796 | 1.13 (0.77 - 1.68)  *P*=0.532 | 1.16 (0.78 - 1.70)  *P*=0.465 |
|  |  |  |  |
| Vascular Repair Phenotype | (ref) | (ref) | (ref) |
| **Acute Kidney Injury** | | | |
| Vascular Injury Phenotype | 3.92 (2.84 - 5.42)  *P*<0.001 | 3.04 (2.12 - 4.35)  *P*<0.001 | 3.06 (2.16 - 4.33)  *P*<0.001 |
|  |  |  |  |
| Dormant Phenotype | 1.23 (0.84 - 1.81)  *P*=0.282 | 1.52 (0.90 - 2.57)  *P*=0.122 | 1.49 (0.89 - 2.47)  *P*=0.128 |
|  |  |  |  |
| Vascular Repair Phenotype | (ref) | (ref) | (ref) |
| **Non-Acute Kidney Injury** | | | |
| Vascular Injury Phenotype | 2.37 (1.53 - 3.67)  *P*<0.001 | 2.02 (1.20 - 3.41)  *P*=0.008 | 2.18 (1.31 - 3.64)  *P*=0.003 |
|  |  |  |  |
| Dormant Phenotype | 0.72 (0.44 - 1.16)  *P*=0.175 | 0.76 (0.40 -1.42)  *P*=0.386 | 0.78 (0.42 - 1.45)  *P*=0.433 |
|  |  |  |  |
| Vascular Repair Phenotype | (ref) | (ref) | (ref) |

Type 3 P-value was <0.01 for the interaction term between AKI and the relationship between clusters and all cause mortality.

**Adjusted**: age, gender, race, ethnicity, body mass index, ever smoker, baseline heart failure, baseline chronic lung disease, baseline chronic obstructive pulmonary disease, baseline cardiovascular disease, baseline hypertension, baseline chronic kidney disease, baseline eGFR, baseline diabetes, admission to the intensive care unit during index hospitalization, diagnosis of sepsis at index hospitalization, vasopressor use during index hospitalization, angiotensin-converting enzymeinhibitor, angiotensin receptor blockers, diuretics, and non-steroidal anti-inflammatory drugs, center, serum creatinine at 3 month (to account for biomarker clearance), proteinuria at 3 month

**Parsimonious Model:** age, gender, race, ethnicity, baseline heart failure, baseline chronic obstructive pulmonary disease, baseline cardiovascular disease, baseline hypertension, baseline diabetes, baseline chronic kidney disease, baseline eGFR, admission to the intensive care unit during index hospitalization, diagnosis of sepsis at index hospitalization, center, serum creatinine at 3 months, proteinuria at 3 month
